# Heterogeneous nanofluids: natural convection heat transfer enhancement

**DOI:** 10.1186/1556-276X-6-222

**Published:** 2011-03-15

**Authors:** Fakhreddine Segni Oueslati, Rachid Bennacer

**Affiliations:** 1 LETTM, Dept de physique, FST Campus Universitaire 2092 El Manar Tunis, Tunisie; 2 ENS-Cachan Dpt GC/LMT, 61, Av du Président Wilson 94235 Cachan Cedex, France

## Abstract

Convective heat transfer using different nanofluid types is investigated. The domain is differentially heated and nanofluids are treated as heterogeneous mixtures with weak solutal diffusivity and possible Soret separation. Owing to the pronounced Soret effect of these materials in combination with a considerable solutal expansion, the resulting solutal buoyancy forces could be significant and interact with the initial thermal convection. A modified formulation taking into account the thermal conductivity, viscosity versus nanofluids type and concentration and the spatial heterogeneous concentration induced by the Soret effect is presented. The obtained results, by solving numerically the full governing equations, are found to be in good agreement with the developed solution based on the scale analysis approach. The resulting convective flows are found to be dependent on the local particle concentration *φ *and the corresponding solutal to thermal buoyancy ratio *N*. The induced nanofluid heterogeneity showed a significant heat transfer modification. The heat transfer in natural convection increases with nanoparticle concentration but remains less than the enhancement previously underlined in forced convection case.

## Introduction

The existence of convection in double-diffusive systems, in which heat and salt diffuse at a different rate, was first recognised in the late 1950 s. Since then, this phenomenon has been studied extensively due to the fact that its importance has been recognised in many fields such as geophysics, astrophysics, ocean physics and industrial processes [1-4]. The first study concerning double diffusion in a binary fluid seems to be that of Nield [5]. Relying on linear stability theory, the onset of motion in an initially motionless, stable concentration and stratified horizontal fluid layer heated from below was predicted by this author. This cross-effect regarding the Rayleigh-Bénard convection dealing with the bifurcation and the possible change in the critical thresholds (i.e. transitional Rayleigh number from conductive to convective motion) was also considered on the same period by Veronis [6]. All the above studies are concerned with the effect of the regular diffusion of each component (heat and salt) on convection. However, in a wide variety of natural and industrial situations, besides the usual diffusion, cross-diffusion between the two agents may also be important. This phenomenon, known as the Soret effect, has been relatively less studied despite its importance for a fluid layer of a binary mixture (convection and stability). In recent studies, the problem of the double thermo-diffusion effects that occurs under natural convection in fluid or porous media was studied; see for example Bennacer et al. [7]

During the past ten years, a new class of fluids made up of metal nanoparticles in suspension in a liquid, called nanofluids, has appeared. Nanofluids are composed of nanoparticles that (size in general <100 nm) are suspended in a base fluid, as water or an organic solvent [8-10]. The formation of extremely stable colloidal systems with very tiny settling is a characteristic feature of some nanofluids, the stability of the suspension is naturally achieved by electrostatic stabilisation by adjusting the pH [11]. The presence of nanoparticles causes a significant modification of thermal properties of the resulting mixture; in particular, nanofluid viscosity and thermal conductivity increase with particle volume fraction. Although the increase in thermal conductivity is a very important interest, there are also increases in the average temperature of nanofluids compared to that of base fluid and that because of the specific heat of nanofluids, which decreases compared to that of base fluid [12,13]. The abnormal rise of the thermal conductivity in comparison with the pure fluid [14], especially for low particle concentrations, is not totally understood today. Some assumptions are based on particle deposition on the surface resulting in the formation of nano fins [15]. There are a many recent studies that report experimental measurements of thermophysical properties of nanofluids, including specific heat, thermal conductivity and viscosity; some recent reports include [16-19]. There has been great attention in nanofluids generated by a variety of applications, ranging from laser-assisted drug delivery to electronic chip cooling.

Some previous research works were mainly concerned with heat transfer and properties of these fluids, see Choi [20], Eastman et al. [21], Maïga et al. [22] and Wang and Mujumdar [23]. The natural convection of nanofluids deserves more attention in light compared to forced convection [24-26]. Recently, linear stability analysis, employed model incorporates the effects of Brownian motion and thermophoresis, for the onset of natural convection in a horizontal nanofluid layer [27]. For vertical layer it was underlined the existence of an optimal particle volume concentration of 2% [28,29], which maximises heat transfer.

The aim of this article is to study the increase of heat transfer taking into account both the variation of thermal conductivity and viscosity in the governing equations when using nanofluids for different types of metallic particles such as Al2O3, TiO2 and Cu. Indeed, for the modelling is as realistic as possible, we considered the Soret effect and the heterogeneity of concentration due to crossed effect.

### Governing equations

**Figure 1:**
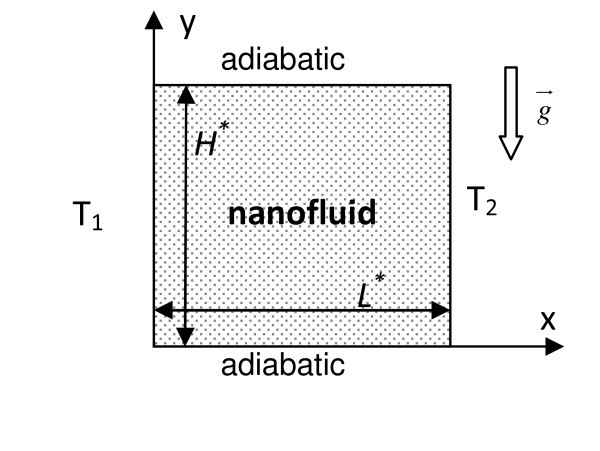
**Physical model and coordinate system**.

In this investigation, convection within a two-dimensional vertical cavity filled by an incompressible Newtonian binary fluid (Figure 1) is studied. All boundaries of the cavity considered are impermeable; the top and bottom boundaries are assumed adiabatic whilst the other vertical ones are kept at uniform but different constant temperatures. The gravity acts in the negative direction (*y*).

In this study, the heterogeneous nanofluid is considered and induced by the Soret-Ludwig effect. The nanofluid (binary mixture with diffusion coeffeicient *D *, see [30]) is modelled as an incompressible fluid possessing an initial uniform particle concentration  and constant physical properties except for the density, which varies with temperature and concentration according to the Boussinesq's approximation as follows:(1)

where *ρ*_0 _is the reference fluid density at temperature and concentration , *β*_T _and *β*_S _are, respectively, the thermal and solutal expansion coefficients, respectively. The microscopic mass flux, taking into account the Soret effect, is given by:(2)

the Soret effect is taken into account if *α *= 1, or ignored if *a *= 0.

The derivation of the coupled governing equations, under their dimensionless form, has been based on the reference quantities for length, velocity, temperature and concentration differences given by cavity height H*, *υ*/H*,  and .

The dimensionless variables (without *) are as follows:

The dimensionless governing equations for, respectively, mass, momentum, energy and concentration are written as:(3)(4)(5)(6)

The heat transfer is characterised by the Nusselt number, which is based on the reference diffusive heat flux: *q*_ref _= *λ*_f _ΔT*/H*

With the exception of the cavity aspect ratio that does not appear explicitly in the equations, but remains indeed a key parameter of the problem, one can notice that the present problem is governed by the thermal Rayleigh number, *R*_T_, the solutal to thermal buoyancy ratio, *N*, the Prandtl number, *Pr*, the Lewis number, *Le *and *a *(for Soret effect occurrence). These parameters are defined as:(7)

It is noted that the thermal coefficient, *β*_T_, is usually a positive quantity. On the other hand, the solutal coefficient *β*_S _can be positive (*N *> 0) or negative (*N *< 0). For *N *> 0, the thermal and solutal boundary forces are both destabilizing, i.e. the two buoyancy components make aiding contributions, whilst for *N *< 0, they make opposing contributions. In the present nanofluide study we have weak concentration but strong buoyancy forces wich is similar to the classical binary mixtures [31].

The controlling thermo-physical properties are the nanofluid to base fluid ratio of thermal conductivity *λ*^r ^= *λ*_nf_/*λ*_f_, and viscosity ratio *μ*^r ^= *μ*_nf_/*μ*_f_. These characteristics are functions of the nanofluid mixture used and furthermore, space dependent due to the possible heterogeneity of nanoparticles concentration. The subscripts f, nf and r refer, respectively, to the base fluid, the nanofluid (effective properties) and relative nanofluid/base fluid ratio of the physical quantity under consideration

The dimensionless thermal, concentration and hydrodynamic boundary conditions are as follows:(8-a)(8-b)(8-c)

The local heat (mass) transfer on the wall is characterised by the local Nusselt (Sherwood) number defined as:(9)

The average number along the active wall is given by  (*M *= *Nu *or *Sh*).

In the above equations, *Nu *represents, as usual, the heat transfers across the walls of the cavity resulting from the combined action of convection and conduction. However, because the walls of the cavity are impermeable, *Sh *does not have its usual significance. Here, it is rather related to the concentration distribution within the cavity induced by the Soret effect (taken into account for *α *= 1, or ignored, i.e. *a *= 0) and by natural convection.

### Numerical method and validation

In order to numerically solve the governing equations, a control volume approach is used. Central differences are used to approximate the advection-diffusion terms, i.e. the scheme is second-order accurate in space. By spatial integration over control volumes, the governing equations are converted into a system of algebraic equations. The latter are solved by a line-by-line iterative method, which is combined with a sweeping technique over the integration domain along *x*- and *y*-axes and a tri-diagonal matrix inversion algorithm. The SIMPLER algorithm is employed to solve the equations in a form of primitive variables. Non-uniform grids are used in the program, allowing fine grid spacing near the two horizontal walls. The convergence criteria are based on the conservation of mass, momentum, energy and species, and this is on both global and local basis.

**Figure 2:**
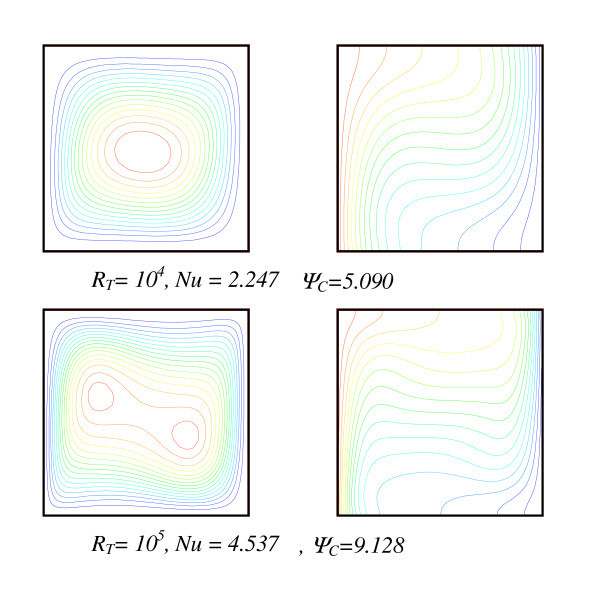
**Dynamic and thermal fields in the case of classical natural convection (without particles and Soret effect, *Pr *= 0.71)**.

Primarily considered a restrictive case of such a model that is the classical natural convection case for which, both influence due to particle volume fraction and Soret effect are considered negligible. Figure 2 shows the thermal and flow structure as obtained for two particular Rayleigh numbers, *R*_T _= 10^4 ^and 10^5^, the values of the stream function at the cavity centre are also given for comparison. Such a structure appears 'conventional' and similar to results reported in the literature [32,33].

A comparison of our numerical data with results from the literature and for these test cases is shown in Table 1. The agreement between our results and others can be qualified as quite satisfactory since the relative maximum deviation was found to be 4%. It is worth noting that due to a clear lack of nanofluids data, it was not possible to validate our mathematical model against experimental data for the specific case of natural convection using nanofluids in a cavity. We do firmly believe that the above agreement may give a confident assessment regarding our mathematical modelling as well as the numerical method adopted.

**Table 1:** Flow intensity in the centre of the cavity versus literature results (*Pr *= 0.7, *A *= 1, *φ *= 0, *Sr *= 0, *N *= 0)

Authors:		Our results	**Leal et al. **[40]	**Sai et al. **[41]	**de Vahl Davis **[33]
					
*R*_T_					
10^3^	*Ψ*_C_	1.136	1.175	-	1.174
	
	*Nu*_moy_	1.117	1.118	1.130	1.117

10^4^	*Ψ*_C_	5.000	5.073	-	5.071
	
	*Nu*_moy_	2.246	2.248	2.289	2.238

10^5^	*Ψ*_C_	9.010	9.112	-	9.111
	
	*Nu*_moy_	4.533	4.562	4.687	4.509

To ensure that the results are mesh-size independent, different non-uniform *n_y _*× *n_x _*meshes (where *n_y _*and *n_x _*represent, respectively, the node numbers in the vertical and horizontal directions), namely 41^2 ^and 81^2^, were thoroughly tested. The difference between results given by those grids was less than 1% for *Nu*, *Sh *and Ψ_c _numbers. Hence, most of the calculations presented in this article were performed using an *n_y _*× *n_x _*= 61^2 ^grid. Such a grid system possesses very fine meshes near all boundaries. The solution was carried out for a validation test case with *Pr *= 0.71 in a narrow channel flow for a range of controlling parameters. The converged solution achieved with all absolute residues of the governing equations is less than 10^-7^. All numerical results presented hereafter are obtained with parameters *A *= 1, *Pr *= 6.2, *Le *= 3 and *Sr *= 2%.

With regard to the effective nanofluid properties, they were evaluated using the following classical relations already known for a two-phase mixture. In the following equations, *p *and *φ*, refer to the particles and particle volume fraction, respectively. The effective density and specific heat of the nanofluid can be estimated on the physical principle of the mixture rule as:(10-a)(10-b)

The viscosity of the nanofluid can be estimated with the existing relations for the two-phase mixture. Drew and Passman [34] introduced Einstein's formula for evaluating the effective viscosity of fluids containing a dilute suspension of small rigid spherical particles, as follows:(11)

This formula is restricted for low particle volume fraction, under 5%. Brinkman [35] proposed the following extension to the Einstein's formula:(12)

Many other relations of effective viscosity of two-phase mixtures exist in the literature. Each relation has its own limitation and application. Some complex behaviour of nanofluids has also been observed by Keblinski et al. [36]. Unfortunately, results reveal that Brinkman's formula underestimates the few experimental data present in literature. In this study, we choose the following polynomial approximation based on experimental data [16,17,37], for water-Al_2_O_3 _nanofluid):(13)

Many experimental researches focussed on nanofluids thermal conductivity, but all of them get different results for the same nanofluid, because of various other parameters influencing this thermal property (concentration, shape and size of particles, dispersants used and particle agglomeration). In this study, we have adopted the Hamilton and Crosser's [38] formula in the case of spherical particles:(14)

## Results and discussion

**Figure 3:**
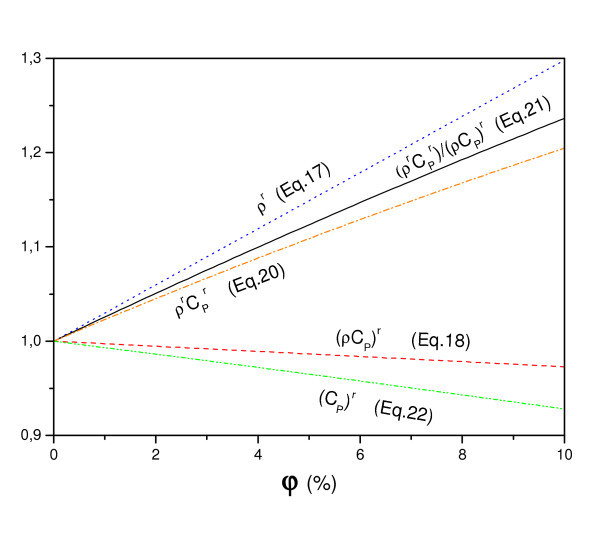
**Specific heat capacity versus nanoparticle concentration (Al_2_O_3_)**.

In this article, despite the lack of experimental results, we use the relative specific heat capacity (*ρC_p_*)^r^, which is the most realistic in the physical sense that the relative density, which multiplies the relative specific heat (*ρ*^r^)(*C_p_*)^r ^which is used by several authors. Indeed, this differentiation is crucial since it greatly affects the results, which is illustrated in Figure 3a. Indeed, the comparison clearly shows that the relative specific heat capacity (*ρ*^r^)(*C_p_*)^r ^continues to grow with the particle fraction of nanofluid, in the case of classical formulation, when it decreases slightly for (*ρC_p_*)^r^.

**Figure 4:**
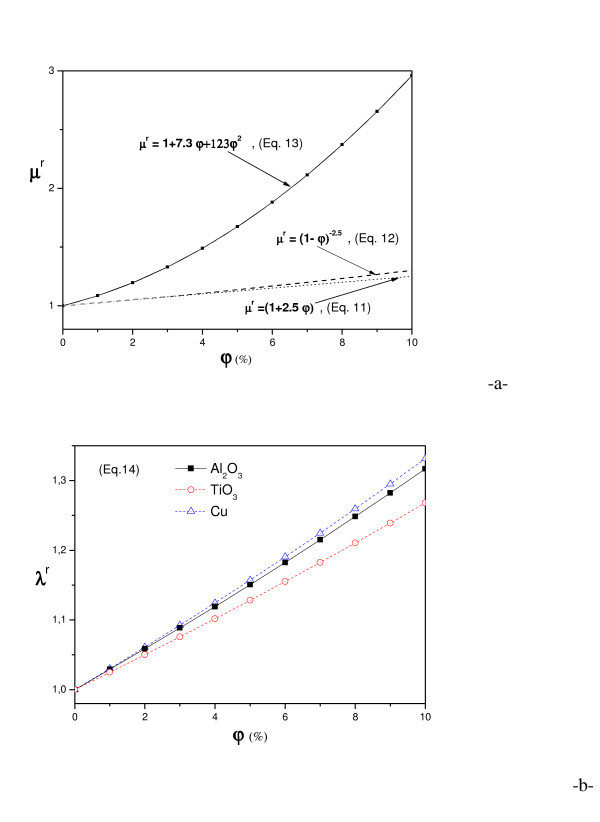
**Relative viscosity (a), thermal conductivity (b) and specific heat capacity (c) versus nanoparticle concentration for different kind of particles**.

As mentioned before, both viscosity and thermal conductivity increase and specific heat capacity decreases with particle concentration (Figure 4).

Based on the definition of the Nusselt number (Equation 7), the heat transfer in the case of homogeneous nanofluid is given by:(15)

where

 where for φ = 0 it recovers the pure fluid case  and the relative nanofluid heat transfer is given by:(16)

This expression illustrates well the evolution of the relative natural convection heat transfer to the pure fluid case. The second group functions only of the particle volume fraction and the relative nanoparticles-to-base fluid viscosity and conductivity (see Equations 13 and 14), and the relative density and heat capacity are given below:(17)(18)

Let's define Δ*ρ*^r ^= (*ρ*_f _- *ρ*_P_)/*ρ*_f _and (19)(20)(21)

and(22)

**Figure 5:**
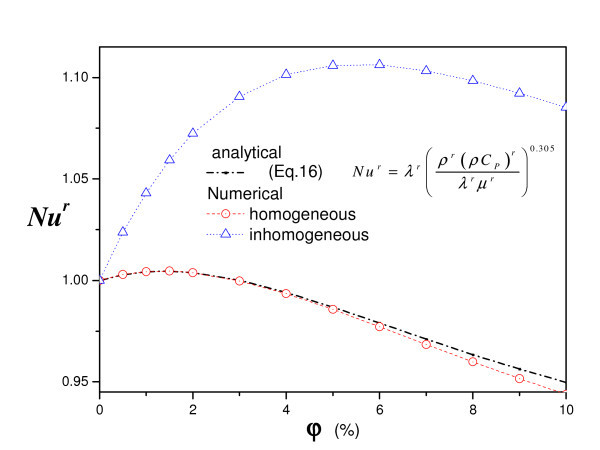
**Nanoparticle fraction effect on heat transfer (*a *= 0, *A *= 1, *R*_T _= 10^5^)**.

The effect of the particle volume fraction on the heat transfer is shown on Figure 5. The same figure exposed a comparison between the classical homogeneous nanofluid model and the heterogeneous nanofluid model. We note, for both homogeneous and heterogeneous as well as the analytical solution, there is a maximum particles concentration above which the heat transfer begins to decrease. In fact the increase of nanofluid viscosity increases the friction, so the flow rate decreases which in turn induces a diminution of heat transfer. On the other hand, an increase of nanofluid thermal conductivity would necessarily enhance the heat transfer. So, it is important to discuss which of these two effects influences most the heat transfer?

Figure 5 shows also, for the classical homogeneous nanofluid model case, that the numerical and analytical results are in good agreement and the maximum Nusselt is reached for particle volume fraction of 2%. Nevertheless, for the case of the heterogeneous fluid model, we can note that the Nusselt is more enhanced and reach a maximum for particle volume fraction of 5%. In fact, the considered thermodiffusion affects clearly the heat transfer and the flow.

When the Soret effect is considered, the nanoparticle concentration within the fluid is spatial dependant (heterogeneous fluid). Such heterogeneity induces a strong non-linear effect as the conductivity, viscosity and heat capacity and solutal buoyancy became spatial-dependant. This explains the strong coupling between the flow, the heat transfer (dependent on the flow and local thermal conductivity) and the concentration which, indeed, is also dependent on both the flow and thermal fields.

**Figure 6:**
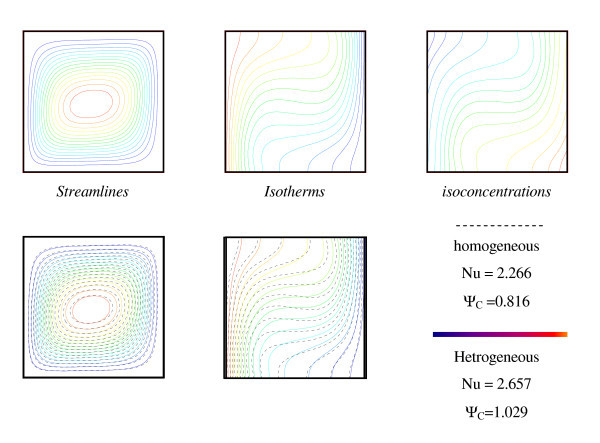
**Dynamic, thermal and concentration fields for homogeneous (plotted by dashed lines) and heterogeneous (plotted by solid lines) cases (*R*_T _= 10^4^, *Pr *= 6.2, *Le *= 3, *Sr *= 0.5%, *φ *= 2%, *N *= 1.75)**.

Figure 6 shows a comparison between homogeneous (plotted by dashed lines) and heterogeneous (plotted by solid lines) cases on streamlines (on the left), isotherms (at the middle) and isoconcentrations (on the right) using the same nanoparticles Al_2_O_3_. The figure demonstrates that a single circulation cell is formed in the clockwise direction for all values of Rayleigh numbers. One can observe that the separation caused by the Soret effect clearly shows the importance of the heterogeneity of the nanoparticle concentration in the cavity. Such a spatial heterogeneity causes, in turn, a relatively important modification of the thermal field, which can modify the heat transfer rate by as much as 10%. It is worth noting that many previous results do not take into account the buoyancy forces effect caused by this heterogeneous distribution of particle concentration. Our results from Figure 6 obviously show that such heterogeneity of nanoparticle concentration induces extra buoyancy forces and would modify the momentum equilibrium. Also, Figure 6 illustrates an example of the resulting dynamic, thermal and species fields as well as the important changes related to the adding temperature and concentration effects.

**Figure 7:**
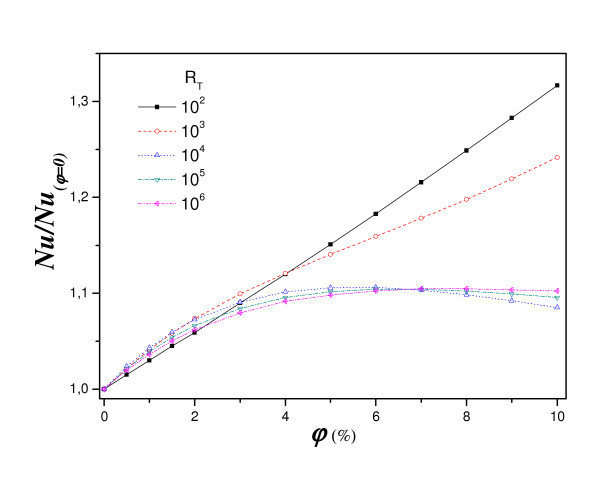
**Effect of nanofluid concentration on relative heat transfer for different *R*_T _(*a *= 1, *A *= 1, *Sr *= 2%, *Pr *= 6.2 and *Le *= 3)**.

The effect of the flow intensity on the optimum value of particle volume fraction observed previously is illustrated on Figure 7. For comparison and discussion purpose, the reference *Nu *for the base fluid is, i.e. fluid without particles, (φ = 0). As usual, *Nu *increases with *R*_T_. The variation of the relative Nusselt number *Nu*^r ^(nanofluid to base fluid) with respect to the particle volume fraction for different *R*_T _is represented in Figure 7. The relative Nusselt number increases in the diffusive regime (low Rayleigh number, *R*_T _< 10^3^) as it is directly dependent on the apparent thermal conductivity. The relative heat transfer (i.e. nanofluid to base fluid) illustrates a decrease for higher Rayleigh number and is a direct consequence of the reference increase illustrated by Figure 7. These results show that the heat transfer is mainly conductive for low value of *R*_T_. For intermediate to high values of *R*_T_, *R*_T _= 10^4^, 10^5 ^and 10^6^, heat transfer first increases with particle volume fraction up to nearly (φ = 5% for *R*_T _= 10^4^, φ = 6% for *R*_T _= 10^5^, φ = 7% for *R*_T _= 10^6^) and then decreases with increasing particle fraction. Such a result for a 'homogeneous' fluid is considered as the reference, based on which we present the relative increase with the concentration for different *R*_T_. The heat transfer increases with increasing particle volume fraction in a monotonic manner for low Rayleigh numbers because of the increase of the fluid thermal conductivity-as the heat transfer mechanism is mainly conduction.

It should be noted that the increase of heat transfer does not exceed 5%. It is worth mentioning that there exists a major difference between the cases of natural convection and forced convection as analysed by others authors, see for example [39]. Such a difference can be explained by the fact that in this study, the flow is not imposed, and hence appears to be more sensitive to a change of the fluid viscosity. The buoyancy strength is governed by the heating conditions imposed so that the intensity of the flow then decreases with increasing viscosity effect.

### Nanoparticle type effect

**Figure 8:**
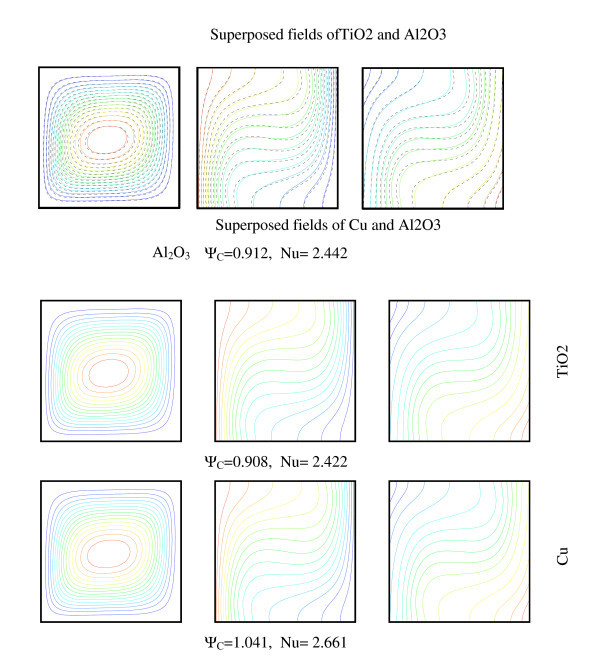
**Dynamic, thermal and species fields for different nature of nanoparticle (*R*_T _= 10^4^, *φ *= 2%, *Pr *= 6.2, *Sr *= 2% and *Le *= 3)**.

**Figure 9:**
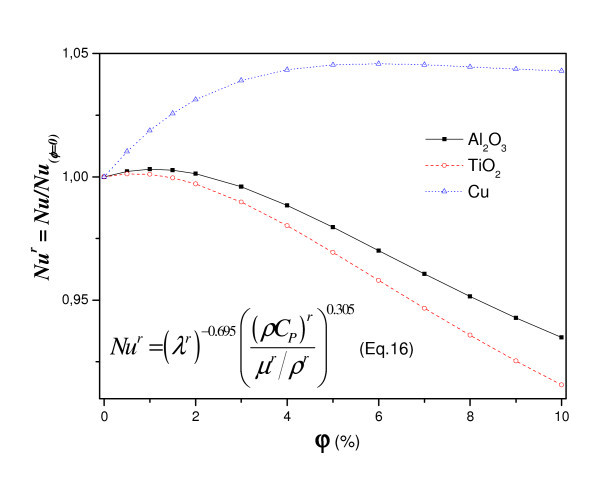
**Nanoparticle fraction effect on heat transfer for different kind of particle: homogeneous case (*a *= 0, *A *= 1, *R*_T _= 10^5^)**.

**Figure 10:**
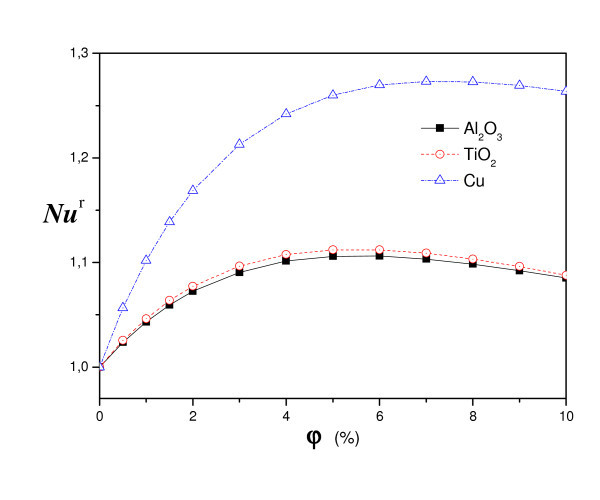
**Effect of nature of nanoparticle on the nanofluid heat transfer: heterogeneous case (*R*_T _= 10^4^, *Pr *= 6.2, *Sr *= 2% and *Le *= 3)**.

**Figure 11:**
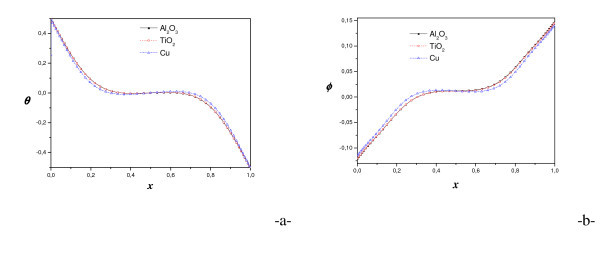
**Temperature (a) and concentration (b) on the horizontal mid-plan (*R*_T _= 10^4^, *φ *= 2%, *Le *= 3 and *Sr *= 2%)**.

Figure 8 presents the comparison of streamlines and isotherms using different nanofluids: TiO_2-_water, Al_2_O_3_-water and Cu-water for *R*_T _= 10^4^. However, we varied the Rayleigh number for different types of nanoparticles, from diffusive state to convection state. For all nanofluids, a single cell movement was observed in a clockwise direction. The values of the maximum stream function show that the intensity of flow is higher for Cu-water than that of TiO_2_-water and Al_2_O_3_-water. Hence, in the case of nanofluid heterogeneous solutal forces are in addition to heat one. The importance of solutal gradients, which differs from one type of nanofluid to another, directly affects the dynamic state and heat transfer (illustrated by figure 9 and 10). Indeed, Figure 11 presents the temperatures (a) and concentrations (b) in the middle horizontal plane of the square enclosure, for different nanofluids (*R*_T _= 10^4^, *Pr *= 6.2, *Le *= 3 and *Sr *= 2%), illustrates the distinction of each type. From superposed streamlines and isotherms of both TiO_2_-water and Al_2_O_3_-water nanofluids, we find that the dynamic and thermal fields are similar. This reproaches qualitative aspects explained by the fact that the values of thermophysical properties of TiO_2_-water and Al_2_O_3_-water are comparable. In opposition, this is not the case for the other two nanofluids Cu-water and Al_2_O_3_-water, which the isotherms and the streamlines show that the distributions are very distinct.

Figure 9 shows the variation of relative Nusselt number, according to analytic approach (Equation 16) with volume fraction using different nanoparticles. We can note that the heat transfer increases with increasing the volume fraction for all nanofluids. For the three nanoparticles one notices the existence of a maximum, which is achieved by increasing the concentration, beyond which the transfer begins to decrease. This finding is valid for Al_2_O_3_-water and TiO_2_-water but not for Cu-water. Indeed, the increase of thermophysical properties as a function of the nanoparticles, namely thermal conductivity, viscosity and specific heat capacity, affects the heat transfer and flow. So, increasing the viscosity with the nanoparticles is exacerbating the friction that causes a decrease in heat transfer. But in the case of Cu, which provides thermal conductivity and density that increases remarkably with the nanoparticles which outweighs the increase in the viscous effect and the specific heat capacity that decreases with the nanoparticles.

We present on Figure 10 the variation of mean Nusselt number with volume fraction using different nanoparticles and different values of Rayleigh number. Results are presented for the case (*R*_T _= 10^4^, *Pr *= 6.2, *Le *= 3 and *Sr *= 2%). The figure shows that the heat transfer increases about monotonically with increasing the volume fraction for all Rayleigh numbers and nanofluids. For the three nanoparticles one notices the existence of a maximum, which is achieved by increasing the concentration, beyond which the transfer begins to decrease, but this maximum differs for Cu (7%), Al_2_O_3 _(6%) and TiO_2 _(5%). The lowest heat transfer was obtained for TiO_2_-water in view of the fact that TiO_2 _has the lowest value of thermal conductivity compared to Cu and Al_2_O_3_. However, the difference in the values of Al_2_O_3 _and TiO_2 _is negligible compared to the value of Cu. The thermal conductivity of TiO_2 _is roughly one fifty of Cu. Yet, a unique property of Al_2_O_3 _is its high specific heat compared to Cu and TiO_2_. The Cu nanoparticles have high values of thermal diffusivity and, thus, this reduces temperature gradients which will affect the performance of Cu nanoparticles. As volume fraction of nanoparticles increases, difference for mean Nusselt number becomes larger especially at higher Rayleigh numbers due to increasing of domination of convection mode of heat transfer. In fact, the temperature gradients grow to be more pronounced, which is illustrate in Figure 11a: the temperature along the middle plane of the square enclosure using different nanofluids for *Ra *= 10^4^, *Pr *= 6.2, *Le *= 3 and Soret coefficient *Sr *= 2%.

**Figure 12:**
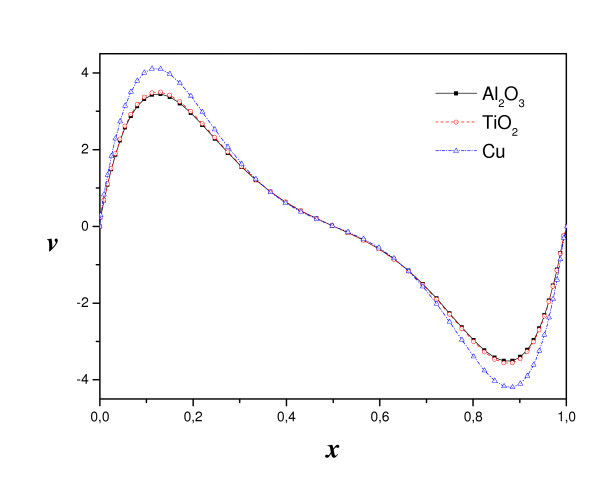
**Vertical velocity on the horizontal mid-plan (*R*_T _= 10^4^, *φ *= 2%, *Le *= 3 and *Sr *= 2%)**.

The vertical velocity along the middle plane of the square enclosure using different nanofluids (for *R*_T _= 10^4^, *Pr *= 6.2, *Le *= 3 and *Sr *= 2%) is shown on Figure 12. Due to the floating flow inside the enclosure, the velocity shows a parabolic variation near the isothermal walls. The vertical velocity is susceptible to the nature of nanoparticles where two types of nanoparticles (Al_2_O_3 _and TiO_2_) show similar vertical velocity but the third (Cu) is so different. This is explained in Equation 16 where the Brinkman formula shows that the viscosity of the nanofluid is only sensitive to the volume fraction of particles and not influenced by the type of nanoparticles and the expression of the buoyancy ration which is a function of the mass expansion coefficient that depends on the density of the nature of the particle. Indeed, the mass buoyancy force, in addition to the thermal buoyancy force, intensified the flow. Even then, the vertical velocity of nanofluid is higher than that of pure fluid. It means that particle suspension affects the flow field. The flow velocity is almost zero around the centre of the cavity. The profile also gives idea on flow rotation direction.

## Conclusion

The effect of using different nanofluids on the thermal and dynamic fields of natural convection in a differentially heated square cavity was studied numerically. Indeed, the results revealed that one type of nanofluid is a key factor for improving heat transfer. The highest values are obtained when using Cu nanoparticles. However, increasing the value of the Rayleigh number is growing the heat transfer. Moreover, the results show the influence due to competing effects between nanoparticles and thermal dynamics, and we identified the flow control parameters for different currents. The results also confirmed that the character of the natural convection directly affects a significant increase in heat transfer with the concentration of particles. Nevertheless the percentage of particle nature greatly affects the heat transfer and fluid flow.

The crossover Soret effect, which is the origin of the spatial distribution of nanoparticles concentrations, and its influence on heat transfer and flow field were studied. The percentage of the optimal nanoparticles concentration that maximises heat transfer was found and it is related to the kind of particle used.

The estimated Soret coefficient was supposed in this study not depending on the nanoparticles but we underline that molecular size and the electrical charges could modify the value of such coefficient and experimental work is necessary to go through this question.

## Abbreviations

### List of symbols

*A*: Aspect ratio of the enclosure, = *L*/*H*; *C*: Concentration; *C_p_*: Specific heat; *D*: Mass diffusivity; *D*_T_: Thermal mass diffusivity; *g*: Gravitational acceleration; *H*: Height of the enclosure; *L*: Width of the cavity; *Le*: Lewis number, = *α*/*D*; *N*: Buoyancy ratio, *β*_S_ΔC*/*β*_T_ΔT*; *Nu*: Nusselt number, Equation 7; *p*: Dimensionless pressure, = p*H/*ρα*; *Pr*: Prandtl number, υ/*α*; *R*_T_: Thermal Rayleigh number, = *gβ*_T _ΔT*H^3 ^*ρC_p_*/υ*α*; *R*_s_: Solutal Rayleigh number, = *gβ*_S _ΔS*H^3 ^*ρC_p_*/υ*λ*; *Sc*: Schmidt nmber = υ/*D*; *Sh*: Sherwood number, Equation 7; *S_r_*: Soret coefficient = *D*_T_/*D*; ΔT*: Characteristic temperature difference; ΔC*: Characteristic concentration difference, ; (*x*, *y*): Dimensionless coordinate system, x*/*H*, y*/*H*; (*u*, *v*): Dimensionless velocity components, u*/(*υ*/*H*), v*/(*υ*/*H*);

### Greek symbols

*α*: Thermal diffusivity, *λ*/(*ρC_p_*); *β*_s_: Solutal expansion coefficient; *β*_T_: Thermal expansion coefficient; *θ*: Dimensionless temperature, ; *ϕ*: Dimensionless concentration, ; *φ*: Particle volume fraction; *λ*: Fluid thermal conductivity; *μ*: Dynamic viscosity; *υ*: Kinematic viscosity; *ρ*: Fluid density; Ψ: Stream function;

### Superscripts

*r*: Nanofluid to base fluid ratio; *: Dimensional variable;

### Subscripts

C: Center; S: Solutal; nf: Nanofluid; f: Base fluid; p: Particle; T: Temperature; 0: Reference state

## Competing interests

The authors declare that they have no competing interests.

## Authors' contributions

FS carried out the numerical simulation studies, participated in the code modification and drafted the manuscript. RB carried out the code development and conceived of the study.
